# MELD 3.0 Score and Time to Discharge After Pericardiectomy: A Competing Risks Analysis in Tuberculous Constrictive Pericarditis

**DOI:** 10.3390/jcm15156118

**Published:** 2026-08-06

**Authors:** Xiaoqiang Gao, Shuzhen Wang, Xin Zhang, Jing Guo, Kunyue Tan, Hong Liu, Wenqian Wu, Feng Xiong

**Affiliations:** 1 Department of Cardiovascular Medicine, The Third People’s Hospital of Chengdu, Chengdu 610031, China; gaoxiaoq1993@163.com (X.G.); zhenzhen8304@163.com (S.W.); 15181965710@163.com (J.G.); 19182174609@163.com (K.T.); liuhong20073721@sina.com (H.L.); 2 Department of Ultrasound Medicine, Union Hospital, Tongji Medical College, Huazhong University of Science and Technology, Wuhan 430022, China; zxin1217@163.com; 3 Hubei Province Clinical Research Center for Medical Imaging, Wuhan 430022, China; 4 Hubei Province Key Laboratory of Molecular Imaging, Wuhan 430022, China

**Keywords:** constrictive pericarditis, tuberculous pericarditis, pericardiectomy, competing risk, hospital discharge, model for end-stage liver disease

## Abstract

**Background/Objectives**: Tuberculous constrictive pericarditis (TCP) is characterized by “backward failure” and systemic venous congestion, leading to secondary multi-organ dysfunction. The Model for End-Stage Liver Disease (MELD) 3.0 score assesses liver, renal, and nutritional status, yet its prognostic utility in TCP remains undefined. This study aimed to evaluate the preoperative MELD 3.0 score as a predictor of time to discharge in patients undergoing pericardiectomy for TCP. **Methods**: We analyzed 190 patients undergoing pericardiectomy for TCP between 2018 and 2024. The association between preoperative MELD 3.0 scores and time to discharge (ICU, postoperative, and total) was assessed using Fine–Gray competing risks regression to account for in-hospital mortality. Models were adjusted for age, albumin, and anti-tuberculosis therapy. **Results**: Each unit increase in MELD 3.0 was associated with higher operative mortality (OR 1.21, *p* = 0.013). In multivariable analysis, a high MELD 3.0 score (>median 9.6) was independently associated with a lower probability of discharge (i.e., delayed discharge) for total hospital stay (subdistribution hazard ratio [sHR] 0.606, 95% CI 0.453–0.811), postoperative stay (sHR 0.665), and ICU stay (sHR 0.658). Preoperative albumin was also a strong predictor of faster discharge. **Conclusions**: The preoperative MELD 3.0 score independently predicts delayed hospital discharge after pericardiectomy for TCP, even when accounting for mortality risk. It serves as a pragmatic tool for quantifying the systemic burden of congestion and stratifying perioperative risk.

## 1. Introduction

Tuberculous constrictive pericarditis (TCP), a sequela of tuberculous pericarditis prevalent in endemic regions, often necessitates pericardiectomy for symptomatic relief [1,2]. Perioperative mortality for this procedure remains substantial, ranging from 2% to 12% [1]. While prior studies have identified predictors such as ejection fraction [3,4] and New York Heart Association (NYHA) functional class [5], TCP is uniquely characterized by “backward failure” [6]. This systemic venous congestion drives progressive organ dysfunction, complicating risk assessment and highlighting the need for tools that quantify congestion-related impairment.

The Model for End-Stage Liver Disease (MELD) score is an extensively validated risk assessment system for evaluating disease severity in patients with end-stage liver cirrhosis [7]. This scoring system integrates serum total bilirubin, creatinine, and the international normalized ratio (INR), serving as an effective marker of multi-organ dysfunction. Consequently, in diseases characterized predominantly by venous congestion, the MELD score correlates with disease severity and predicts clinical outcomes across different risk strata [6].

Notably, the MELD score has demonstrated prognostic value in populations undergoing cardiac surgery, including tricuspid valvuloplasty, pericardiectomy, and left ventricular assist device implantation [6,8,9]. Similarly, its derivative MELD-XI (excluding INR) has been validated in cardiovascular cohorts [6,10,11].

MELD 3.0 represents the latest iteration of the MELD scoring system, proposed by Kim et al. [12]. This model incorporates sex-specific adjustments with differential weighting for males and females, and additionally includes serum albumin and sodium levels to more accurately reflect disease severity. However, current research on the association between MELD 3.0 and cardiovascular disease prognosis remains limited. To address this gap, we adopted time to discharge as the primary endpoint in preference to a mortality-based outcome. Although perioperative mortality after pericardiectomy remains a concern [1], event rates in contemporary series are low—no in-hospital deaths were observed in a recent tuberculous cohort [5], and 90-day mortality was 8.7% in a large single-center study [6]—which limits statistical power and precludes robust multivariable analysis. Time to discharge, by contrast, is an objective and patient-centered measure that integratively reflects the burden of postoperative morbidity, since preoperative organ dysfunction and postoperative complications have consistently translated into prolonged intensive care and hospital stays [5,6]. The endpoint is also well suited to the pathophysiology of TCP, in which chronic systemic venous congestion drives multi-organ dysfunction and recovery after relief of constriction is gradual; the timing of discharge thus captures how quickly the congestive syndrome quantified by the MELD 3.0 score actually resolves. Moreover, because virtually all patients experience this event, statistical efficiency is maximized, while treating in-hospital death as a competing event ensures that mortality is appropriately accounted for rather than ignored. We therefore aimed to determine whether the preoperative MELD 3.0 score predicts delayed hospital discharge after pericardiectomy for TCP. Additionally, we assessed its association with in-hospital mortality and major perioperative complications.

## 2. Materials and Methods

### 2.1. Study Population

This single-center retrospective cohort study enrolled 190 patients who were first hospitalized for tuberculous constrictive pericarditis and underwent pericardiectomy between January 2018 and June 2024. Tuberculous constrictive pericarditis was defined as highly suspected or definitively diagnosed tuberculous pericarditis. The clinical diagnosis was based on established diagnostic criteria [13]:

1. Definite tuberculous pericarditis: identification of *Mycobacterium tuberculosis* on staining or culture of pericardial effusion; and/or demonstration of the bacillus or caseating granulomas on histologic examination of pericardial tissue.
2. Probable tuberculous pericarditis: evidence of pericarditis in a patient with confirmed tuberculosis elsewhere in the body; and/or lymphocytic pericardial exudate with elevated adenosine deaminase (ADA) activity; and/or a good response to anti-tuberculosis therapy.

Inclusion criteria: (1) complete laboratory data for MELD 3.0 calculation; (2) age ≥ 18 years.

Exclusion criteria: (1) previous pericardiectomy; (2) non-tuberculous constrictive pericarditis; (3) missing key outcome data.

### 2.2. Variable Definitions

#### 2.2.1. MELD 3.0 Score

To calculate the preoperative MELD 3.0 score, the first laboratory results after hospitalization were used, applying the established formula by Kim et al. [12]. In accordance with the standard algorithm, lower limits were applied to bilirubin, creatinine, and INR (set to 1), while serum sodium and albumin were constrained to ranges of 125–137 mEq/L and 1.5–3.5 g/dL, respectively.

#### 2.2.2. Definition of Endpoints

The study outcomes were categorized into two types. The first type, analyzed as exploratory endpoints, comprised postoperative major adverse events. Based on clinical importance and event rates, we included the following events occurring during the postoperative hospital stay: all-cause mortality, postoperative acute kidney injury (AKI), and postoperative low cardiac output syndrome (LCOS).

The second and principal type of outcome was time to discharge. Time to discharge was defined as the interval from the date of surgery to the date of hospital discharge and was analyzed in three specific dimensions: (1) time to intensive care unit (ICU) discharge (surgery date to ICU discharge date); (2) postoperative time to discharge (surgery date to hospital discharge date, excluding preoperative hospitalization time); and (3) total time to discharge (hospital admission date to discharge date). All time variables were measured in days.

Discharge decisions followed uniform institutional criteria throughout the study period. ICU discharge required hemodynamic and respiratory stability with no or minimal vasoactive support, minimal chest drainage without active bleeding, and no complications requiring continued intensive monitoring; hospital discharge required satisfactory wound healing, removal of all drains, adequate volume control on oral diuretics without decompensated heart failure, stable hepatic and renal function with tolerated anti-tuberculosis therapy, and completed discharge planning.

#### 2.2.3. Covariates and Confounding Factors

Based on literature and clinical experience, potential confounding factors were pre-specified, including demographic characteristics, nutritional and metabolic indices, hepatic and renal function, cardiac functional status, treatment-related factors, and surgical characteristics (detailed in Table 1). Anti-tuberculosis therapy was defined as receiving ≥2 weeks of standard therapy preoperatively.

### 2.3. Sample Size Estimation

A post-hoc sample size assessment based on events per variable (EPV) demonstrated adequate statistical power. For the primary endpoint (discharge, *n* = 181 events), with 4 pre-specified covariates in the core model, the EPV was 45, well above the recommended threshold of 10.

### 2.4. Statistical Analysis Methods

All analyses were conducted using R (version 4.5.0; R Foundation for Statistical Computing, Vienna, Austria). Continuous variables are presented as mean ± SD or median (IQR) and compared using *t*-tests or Mann–Whitney U tests, as appropriate. Categorical variables are presented as *n* (%) and compared using χ^2^ or Fisher’s exact tests. A two-sided α = 0.05 defined statistical significance.

For the exploratory endpoints (postoperative major adverse events), due to the limited number of events (*n* = 9 for mortality, *n* = 23 for AKI, *n* = 17 for LCOS), only univariable logistic regression analyses were performed.

For the primary analysis of time to discharge, we employed Fine–Gray competing risks models, treating in-hospital death as a competing event. Variable selection followed a two-stage approach: (1) univariable Fine–Gray screening of pre-specified covariates (*p* < 0.10 for inclusion consideration), which identified several candidate variables (preoperative albumin, NYHA class, INR, creatinine, dialysis, diuretic use, concomitant procedures, and total bilirubin); and (2) To construct a parsimonious and clinically interpretable core model while controlling for multicollinearity and adhering to the events per variable (EPV) principle (181 discharge events for 4 planned covariates, EPV = 45), we prioritized variables based on clinical relevance and statistical strength. Age was included a priori as a fundamental demographic confounder. Anti-tuberculosis therapy was retained due to its clinical importance as a key treatment-related factor. Preoperative albumin was selected based on its strong univariable association—it was the strongest predictor among all covariates in univariable Fine–Gray analysis (sHR 2.356, *p* < 0.001)—and its biological plausibility as a marker of nutritional and inflammatory status. Notably, despite albumin being a component of the MELD 3.0 score, multicollinearity assessment using variance inflation factors (VIF) showed no significant collinearity between these two variables (VIF = 1.19 for albumin; all VIF values < 1.2). Although other variables showed univariable associations, they were not included in the core model to avoid multicollinearity and maintain model parsimony, as these variables capture overlapping aspects of disease severity already reflected by the MELD 3.0 score components. Consequently, the final multivariable models were adjusted for age, preoperative albumin, and anti-tuberculosis therapy.

The proportional subdistribution hazards assumption was assessed graphically [log(–log) transformed curves] and via Cox models with time-by-covariate interaction terms; no significant violations were detected. Results are reported as subdistribution hazard ratios (sHR) with 95% confidence intervals (CI) (sHR < 1 indicates slower discharge in the high MELD 3.0 group).

To evaluate the dose–response relationship and to address concerns regarding potential loss of statistical power from dichotomization, we also performed supplementary analyses treating MELD 3.0 as a continuous variable.

Model discrimination was assessed using time-dependent receiver operating characteristic (ROC) curves with corresponding area under the curve (AUC) values at clinically relevant time points. AUC 95% confidence intervals were estimated using 1000 bootstrap resamples. The linear predictor from the multivariable Fine-Gray model was used as the marker for time-dependent ROC analysis.

Sensitivity analyses included: (1) subgroup analysis in patients without anti-tuberculosis treatment (*n* = 51); (2) interaction assessment between MELD 3.0 score and anti-tuberculosis treatment; and (3) Bonferroni correction for three primary outcomes (adjusted α = 0.017).

For visualization, cumulative incidence curves and forest plots were generated.

## 3. Results

### 3.1. Baseline Characteristics

A total of 190 patients who underwent pericardiectomy for tuberculous constrictive pericarditis were included in the analysis. The median age was 52.0 years (interquartile range [IQR], 29.8–64.0), and 67 (35.3%) were female.

Patients were stratified into low and high MELD 3.0 groups (median 9.6; each *n* = 95). As detailed in Table 1, the high-score group exhibited significantly worse profiles across all MELD 3.0 components (all *p* < 0.01), more advanced heart failure, greater diuretic dependence, but less frequent pretreatment with anti-tuberculosis therapy (all *p* < 0.05).

### 3.2. Postoperative Major Adverse Events

During the postoperative hospital stay, there were 9 (4.7%) in-hospital deaths, 23 (12.1%) cases of postoperative acute kidney injury, and 17 (8.9%) cases of postoperative low cardiac output syndrome. Univariable logistic regression analyses (Supplementary Table S1) showed that per 1-unit increase in preoperative MELD 3.0 score (continuous) was associated with higher risk of operative mortality (OR 1.21, 95% CI 1.04–1.41, *p* = 0.013) and postoperative renal failure (OR 1.18, 95% CI 1.03–1.35, *p* = 0.015), but not with postoperative low cardiac output syndrome (OR 1.03, 95% CI 0.92–1.16, *p* = 0.559). Given the limited number of death events (*n* = 9), these analyses are exploratory and hypothesis-generating, and should be interpreted with caution.

### 3.3. Association Between MELD 3.0 Score and Time to Discharge

**Figure 1:**
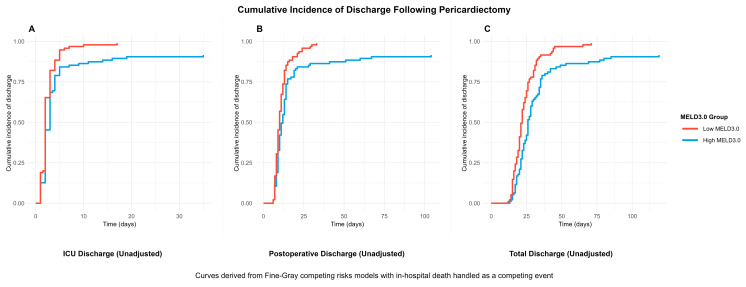
Cumulative incidence of discharge following pericardiectomy, stratified by preoperative MELD 3.0 score. Fine-Gray competing risk curves for (**A**) ICU, (**B**) postoperative, and (**C**) total discharge, treating in-hospital death as the competing event. Curves compare high (blue) versus low (red) MELD 3.0 scores (dichotomized at median 9.6).

The cohort had a median total hospital stay of 23 days (IQR 17–32). Following pericardiectomy, the median postoperative stay was 9 days (IQR 8–13), including a median ICU stay of 2 days (IQR 1–4). As shown in Figure 1, cumulative incidence curves consistently demonstrated that patients in the low MELD 3.0 group experienced faster discharge across all three time-to-event outcomes than the high-score group.

In univariable Fine-Gray competing risks models, a high MELD 3.0 score (dichotomized) was significantly associated with delayed discharge for ICU discharge (sHR 0.681, 95% CI 0.541–0.856, *p* = 0.001), postoperative discharge (sHR 0.636, 95% CI 0.487–0.832, *p* = 0.0009), and total discharge (sHR 0.546, 95% CI 0.410–0.727, *p* < 0.001).

**Figure 2:**
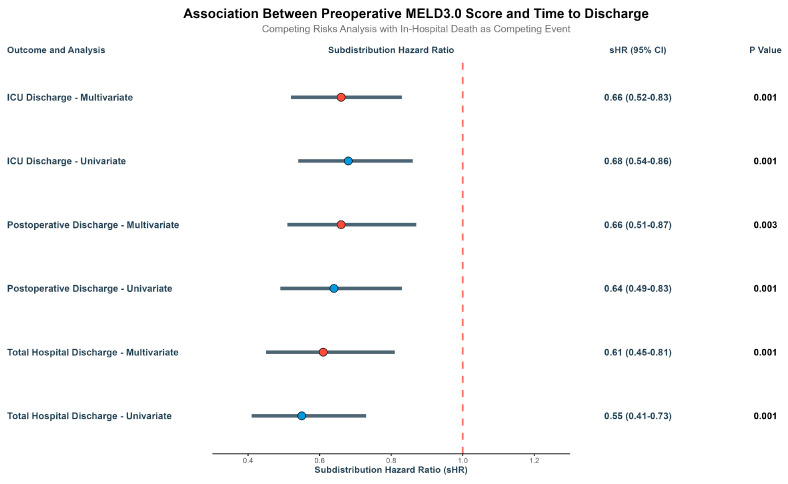
Association between preoperative MELD 3.0 score and time to discharge: univariable and multivariable competing risks analyses. Subdistribution hazard ratios (sHR) shown for high vs. low MELD 3.0 scores across endpoints. Blue points: univariable analysis; Red points: multivariable analysis adjusted for age, albumin, and anti-tuberculosis therapy. Error bars denote 95% CIs. An sHR < 1 indicates delayed discharge.

After multivariable adjustment (Table 2), a high MELD 3.0 score remained independently associated with delayed discharge across all endpoints: total discharge (sHR 0.606, 95% CI 0.453–0.811, *p* = 0.0007), postoperative discharge (sHR 0.665, 95% CI 0.507–0.874, *p* = 0.0034), and ICU discharge (sHR 0.658, 95% CI 0.519–0.834, *p* = 0.0006). Preoperative albumin was a strong independent predictor of faster discharge (Table 2). These associations are visually summarized in Figure 2.

To further confirm the robustness of the primary finding and to evaluate a potential dose–response relationship, we repeated the multivariable analysis treating MELD 3.0 as a continuous variable (Supplementary Table S2). The association remained statistically significant across all three endpoints: total discharge (sHR 0.878 per 1-unit increase, 95% CI 0.831–0.927, *p* < 0.001), postoperative discharge (sHR 0.900, 95% CI 0.858–0.945, *p* < 0.001), and ICU discharge (sHR 0.905, 95% CI 0.865–0.947, *p* < 0.001), confirming a dose–response relationship.

To quantify the discriminative performance of the multivariable model, we calculated time-dependent AUC values at clinically meaningful time points (Supplementary Table S3). From day 21 onward, when 73 patients (38.4%) had been discharged, AUC values consistently exceeded 0.70, indicating good discriminative performance. The peak discriminative ability was observed at 45 days (AUC = 0.742, 95% CI: 0.625–0.853), with 171 patients (90.0%) discharged by this time. Time-dependent ROC curves comparing the 21-day and 45-day time points are shown in Supplementary Figure S1.

### 3.4. Supplemental Analyses and Model Assumptions

Sensitivity analyses supported the robustness of the primary findings (Supplementary Tables S4–S7). In the subgroup of patients without preoperative anti-tuberculosis therapy, the association between high MELD 3.0 score and delayed discharge was attenuated and not statistically significant. There was no significant interaction between MELD 3.0 score and anti-tuberculosis therapy. The associations remained significant after Bonferroni correction for multiple comparisons. Assessment of baseline imbalances confirmed the necessity of adjusting for anti-tuberculosis therapy, age, and albumin in the primary model (Supplementary Figure S2). Graphical evaluation did not indicate major violations of the proportional subdistribution hazards assumption (Supplementary Figures S3–S5, Supplementary Table S7).

## 4. Discussion

In this study, a high preoperative MELD 3.0 score (>median 9.6) was independently associated with delayed discharge across all endpoints after adjusting for mortality (all *p* < 0.01). Notably, preoperative albumin was an even stronger predictor of faster discharge, while anti-tuberculosis therapy did not significantly modify the association (interaction *p* = 0.447). These findings support the potential utility of MELD 3.0 for preoperative risk stratification in this specific patient population and highlight the critical role of nutritional status in postoperative recovery.

The findings of this study are highly congruent with the core pathophysiological feature of constrictive pericarditis—”backward failure” dominated by venous congestion. Under this condition, persistently elevated central venous pressure can lead to progressive hepatic and renal dysfunction as well as nutritional and metabolic disturbances [2,14,15,16]. The MELD 3.0 score integrates parameters reflecting hepatic, renal, nutritional, and homeostatic status. It may therefore serve as a useful quantitative tool for assessing preoperative organ functional reserve and prognosis in such patients.

Our findings extend the previous work by Diaz Soto et al. [6], who demonstrated that the MELD-XI score was an independent predictor of mortality and acute kidney injury following pericardiectomy. Our study contributes to this body of evidence in several ways. First, we evaluated the prognostic value of the updated MELD 3.0 score in this patient population. Compared with MELD-XI, MELD 3.0 theoretically offers a more comprehensive capture of the complex metabolic and neuroendocrine alterations associated with congestive states by incorporating sex, serum albumin, and sodium [12]. Second, we specifically focused on tuberculous constrictive pericarditis, a subgroup facing unique therapeutic challenges (e.g., potential hepatotoxicity of anti-tuberculosis drugs) and epidemiological contexts [17]. Third, methodologically, we used “time to discharge” as the primary endpoint and employed competing risks models to disentangle the risk of delayed discharge from that of in-hospital mortality. The observed association of MELD 3.0 with discharge time complements prior evidence for MELD-XI and mortality, collectively supporting the prognostic utility of MELD-based scores in constrictive pericarditis.

Another key finding of this study is the strong protective association of preoperative serum albumin, whose predictive power in our cohort exceeded that of the MELD 3.0 score itself. This lends empirical support to the prospective inclusion of albumin in the MELD 3.0 calculation. Albumin is more than a simple nutritional marker; it serves as an integrative indicator of systemic inflammation and metabolic homeostasis [18,19,20]. Dynamic decreases in albumin during hospitalization are powerful predictors of infectious complications and worsening clinical outcomes [21]. In our study population, low albumin may reflect persistent inflammation and catabolism secondary to chronic venous congestion, making it a sensitive indicator for predicting postoperative recovery speed.

Finally, our handling of the important clinical factor—anti-tuberculosis therapy—strengthens the reliability of the results. It is known that anti-tuberculosis drugs (e.g., isoniazid, rifampicin, pyrazinamide) carry potential hepatotoxicity and nephrotoxicity [22,23], which could influence the parameters included in the MELD score and confound its prognostic significance. Baseline characteristics differed between the treatment groups in our study (younger but with higher MELD 3.0 scores). Nevertheless, multivariable adjustment and interaction analysis showed that treatment status did not significantly modify the association between MELD 3.0 and time to discharge. This suggests that the prognostic information conveyed by the MELD 3.0 score may remain stable across different treatment backgrounds, even after accounting for potential treatment-related toxicity. This information is likely rooted in organ dysfunction due to venous congestion. This supports its potential utility as a generalizable risk stratification tool.

This risk stratification also has practical implications for perioperative management. The MELD 3.0 score is derived from objective, routinely collected laboratory data [12]. It can therefore be incorporated into the preoperative work-up of patients with tuberculous constrictive pericarditis without additional cost or testing burden, providing a patient-specific estimate of congestion-related organ dysfunction that facilitates individualized risk communication [6]. Patients identified as high risk may benefit from intensified preoperative optimization of modifiable derangements, such as more aggressive decongestion. Given the strong protective association of albumin observed in our cohort, nutritional support may also be warranted, together with heightened postoperative surveillance. This anticipation is empirically grounded. In the pericardiectomy cohort of Diaz Soto et al., increasing MELD and MELD-XI scores were associated with greater postoperative bleeding, transfusion requirements, and renal failure [6]. MELD-XI additionally predicted prolonged mechanical ventilation and longer intensive care and hospital stays [6]. In tuberculous constrictive pericarditis specifically, congestion severity markers—NYHA functional class and central venous pressure—independently predicted postoperative complications, which in turn were the independent predictors of prolonged ICU stay [5]. An elevated preoperative MELD 3.0 score may therefore prompt the surgical team to anticipate greater resource utilization, including blood products, renal support, and ICU capacity. It may also justify intensified monitoring for complications such as acute kidney injury and low cardiac output syndrome, and more guarded counselling regarding the expected pace of recovery. In this way, the MELD 3.0 score serves not merely as a static prognostic label but as a dynamic marker of current illness severity, enabling a proactive rather than reactive approach to perioperative care [6].

The strengths of this study include its focus on a pathophysiologically relevant cohort, providing an evaluation of MELD 3.0 for predicting patient-centered discharge time in this setting, and the rigorous use of competing risks analysis with comprehensive sensitivity testing.

Limitations include its retrospective, single-center design, which may limit generalizability and leave potential for residual confounding. Cardiac biomarkers—particularly troponin T and NT-proBNP—were not systematically collected in this retrospective cohort, limiting our ability to assess their association with prognosis. Similarly, post-tuberculosis changes in other organs, such as pulmonary sequelae, were not systematically documented in this cohort, precluding assessment of their association with prognosis. The limited number of in-hospital deaths (*n* = 9) precluded multivariable adjustment for mortality-related analyses. The observed associations with mortality should therefore be considered exploratory and require confirmation in larger cohorts. The non-significant association in the untreated subgroup (*n* = 51) is likely due to reduced statistical power. Our definition of anti-tuberculosis therapy lacked granularity, and the exploratory analyses of adverse events are hypothesis-generating.

These findings warrant external validation in independent, multicenter cohorts before the MELD 3.0 score can be considered for routine clinical implementation. Until such evidence is available, the score should be regarded as a research tool for risk stratification rather than a basis for clinical decision-making. If validated, future work should focus on integrating MELD 3.0 into clinical risk models and developing targeted management pathways for high-risk patients.

## 5. Conclusions

The preoperative MELD 3.0 score is a robust, independent predictor of delayed discharge after pericardiectomy for tuberculous constrictive pericarditis, even when accounting for the competing risk of death. It pragmatically stratifies preoperative risk by capturing the systemic burden of venous congestion. The stronger predictive value of preoperative albumin underscores the critical role of nutrition and inflammation in recovery.

## Figures and Tables

**Table 1:** Comparison of Baseline Characteristics Between the Low and High MELD 3.0 Groups.

Variable	Overall Population (*n* = 190)	Low MELD 3.0 (*n* = 95)	High MELD 3.0 (*n* = 95)	*p*-Value
Age at surgery (years)	52.0 (29.8, 64.0)	52.0 (33.0, 64.0)	50.0 (25.0, 64.0)	0.43 *
Gender (female)	67 (35.3%)	34 (35.8%)	33 (34.7%)	0.87 ^&^
BMI (kg/m^2^)	22.0 (20.2, 24.3)	22.5 (20.0, 24.6)	22.4 (20.4, 24.2)	0.83 *
MELD score	8.3 (6.8, 10.0)	7.0 (6.4, 8.0)	10.0 (8.9, 11.5)	<0.001 *
MELD 3.0 score	9.6 (8.2, 11.9)	8.2 (7.5, 9.1)	11.9 (10.3, 13.7)	<0.001 *
Total bilirubin (mg/dL)	1.0 (0.7, 1.6)	0.8 (0.6, 1.1)	1.5 (1.0, 2.0)	<0.001 *
Creatinine (mg/dL)	0.8 (0.6, 0.9)	0.7 (0.6, 0.8)	0.8 (0.7, 0.9)	0.006 *
INR	1.1 (1.0, 1.2)	1.0 (1.0, 1.1)	1.2 (1.1, 1.3)	<0.001 *
Serum sodium (mEq/L)	137.0 (136.8, 137.0)	137.0 (137.0, 137.0) ^†^	137.0 (135.5, 137.0) ^†^	<0.001 *
Serum albumin (g/dL)	3.30 (3.02, 3.50)	3.50 (3.50, 3.50) ^†^	3.50 (3.17, 3.50) ^†^	<0.001 *
Creatinine clearance (mL/min)	73.0 (61.9, 87.7)	73.6 (63.0, 91.5)	70.7 (58.2, 86.0)	0.06 *
NYHA class				0.005 ^#^
I	9 (5.5%)	5 (6.3%)	4 (4.8%)	
II	62 (38.0%)	41 (51.2%)	21 (25.3%)	
III	76 (46.6%)	29 (36.3%)	47 (56.6%)	
IV	16 (9.8%)	5 (6.3%)	11 (13.3%)	
LVEF (%)	60 (55,63)	60 (56,64)	59 (54,63)	0.85 *
Medical history				
Diabetes mellitus	16 (8.4%)	8 (8.4%)	8 (8.4%)	1 ^&^
Arterial hypertension	17 (8.9%)	11 (11.6%)	6 (6.3%)	0.20 ^&^
Atrial fibrillation	17 (8.9%)	6 (6.3%)	11 (11.6%)	0.21 ^&^
Chronic Obstructive Pulmonary Disease	21 (11%)	12 (12.6%)	9 (9.5%)	0.49 ^&^
Coronary heart disease	50 (26.5%)	24 (25.3%)	26 (27.7%)	0.71 ^&^
Pre-operative medication/treatment				
Preoperative anti-TB therapy	139 (76.3%)	76 (80%)	63 (66.3%)	0.049 ^&^
Preoperative dialysis	5 (2.6%)	0 (0%)	5 (5.3%)	0.059 ^#^
Preoperative warfarin therapy	56 (29.5%)	24 (25.3%)	32 (33.7%)	0.203 ^&^
Diuretics (30 days before surgery)	153 (80.5%)	68 (71.6%)	85 (89.5%)	0.002 ^&^
Concomitant procedures	21 (11.1%)	7 (8.4%)	14 (13.1%)	0.31 ^&^
CABG	12 (6.3%)	5 (6.0%)	7 (6.5%)	0.88 ^&^
Valve Repair and/or Valve Replacement	5 (2.6%)	1 (1.2%)	4 (3.7%)	0.39 ^#^
Others	7 (3.7%)	2 (2.4%)	5 (4.7%)	0.47 ^#^

Abbreviations: BMI, Body mass index; CABG, coronary artery bypass grafting; INR, international normalized ratio; LVEF, left ventricular ejection fraction; MELD, Model for End-Stage Liver Disease; NYHA, New York Heart Association; TB, tuberculosis. Note: Data are presented as *n* (%) for categorical variables and median (interquartile range) for continuous variables. Statistical symbols: * Calculated using the Mann–Whitney U test. ^&^ Calculated using the Chi-square test. ^#^ Calculated using Fisher’s exact test. ^†^ For serum albumin and serum sodium, values were censored at the MELD 3.0 calculation limits (1.5–3.5 g/dL and 125–137 mEq/L) as per the updated methodology [12], resulting in identical medians but different underlying distributions.

**Table 2:** Multivariable Fine-Gray competing risks regression analysis for time to discharge outcomes.

Outcome	Variable	Comparison	sHR	95% CI	*p*-Value
ICU Discharge	MELD 3.0 score	High vs. Low	0.658	0.519–0.834	0.0006
	Age	Per year increase	1.002	0.996–1.009	0.465
	Preoperative albumin	Per 1 g/dL increase	1.206	0.849–1.714	0.296
	Preoperative anti-TB therapy	Yes vs. No	0.741	0.573–0.959	0.0225
Postoperative Discharge	MELD 3.0 score	High vs. Low	0.665	0.507–0.874	0.0034
	Age	Per year increase	0.995	0.988–1.002	0.185
	Preoperative albumin	Per 1 g/dL increase	1.722	1.127–2.632	0.012
	Preoperative anti-TB therapy	Yes vs. No	0.701	0.525–0.936	0.016
Total Discharge	MELD 3.0 score	High vs. Low	0.606	0.453–0.811	0.0007
	Age	Per year increase	0.993	0.983–1.001	0.093
	Preoperative albumin	Per 1 g/dL increase	2.113	1.385–3.230	0.0005
	Preoperative anti-TB therapy	Yes vs. No	0.736	0.529–1.023	0.068

Abbreviations: CI, Confidence interval; ICU, intensive care unit; MELD, Model for End-Stage Liver Disease; sHR, subdistribution hazard ratio; TB, tuberculosis. Models are adjusted for age, preoperative albumin, and anti-tuberculosis therapy, with in-hospital death as a competing event. sHR, subdistribution hazard ratio; sHR < 1 indicates slower discharge in the high MELD 3.0 group.

## Data Availability

The data presented in this study are available on request from the corresponding author. The data are not publicly available due to privacy and ethical restrictions involving patient medical records.

## Supplementary Materials

The following supporting information can be downloaded at https://www.mdpi.com/article/10.3390/jcm15156118/s1: Figure S1: Time-dependent ROC curves (21 days vs 45 days); Figure S2: Standardized Mean Differences (SMD) for baseline characteristics with notable imbalances between treatment groups; Figure S3: Proportional hazards assumption assessment for ICU discharge; Figure S4: Proportional hazards assumption assessment for postoperative discharge; Figure S5: Proportional hazards assumption assessment for total hospital discharge; Table S1: Univariate associations between preoperative MELD 3.0 score and postoperative major adverse events; Table S2: Multivariable Fine-Gray competing risks regression analysis with MELD 3.0 as a continuous variable; Table S3: Time-dependent AUC values at clinically relevant time points; Table S4: Subgroup analysis: Multivariable competing risks model for time to total hospital discharge in patients without preoperative anti-tuberculosis therapy (*n* = 51); Table S5: Interaction analysis: Association between MELD 3.0 score and total discharge time including interaction term; Table S6. Bonferroni correction for multiple comparisons across three time-to-discharge outcomes; Table S7. Assessment of Proportional Hazards Assumption for Fine-Gray Competing Risks Models.

## Abbreviations

The following abbreviations are used in this manuscript:

ADA	Adenosine Deaminase
CABG	Coronary Artery Bypass Grafting
CI	Confidence Interval
CVP	Central Venous Pressure
EPV	Events Per Variable
ICU	Intensive Care Unit
INR	International Normalized Ratio
IQR	Interquartile Range
LVEF	Left Ventricular Ejection Fraction
MELD	Model for End-Stage Liver Disease
MELD-XI	Model for End-Stage Liver Disease Excluding INR
NYHA	New York Heart Association
OR	Odds Ratio
sHR	Subdistribution Hazard Ratio
TB	Tuberculosis
TCP	Tuberculous Constrictive Pericarditis
