# Supplementary material for: MELD 3.0 Score and Time to Discharge After Pericardiectomy: A Competing Risks Analysis in Tuberculous Constrictive Pericarditis
Source: J Clin Med. 2026 Aug 6;15(15):6118. doi: 10.3390/jcm15156118 (PMC13466852; doi:10.3390/jcm15156118)
Supplement: Supplementary file 1 [file jcm-15-06118-s001.zip › jcm-4424810-supplementary.pdf]

Figure S1. Time-dependent ROC curves (21 days vs 45 days)

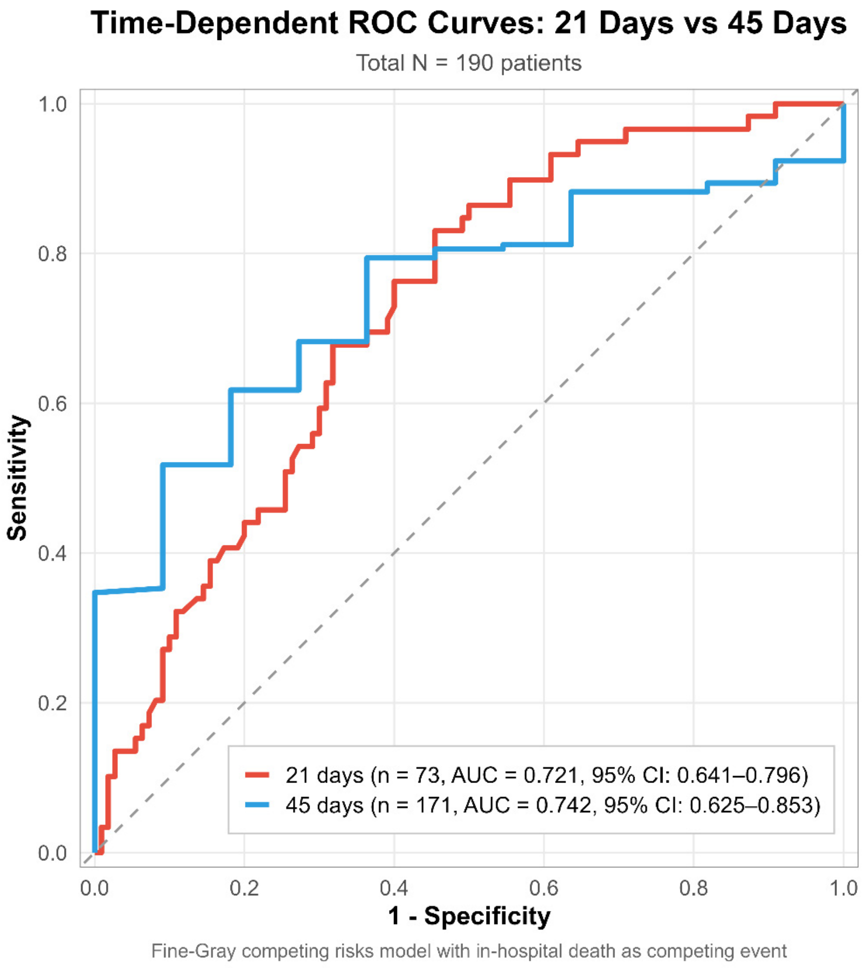

Time-dependent ROC curves for the total discharge model at 21 days (red) and 45 days (blue). The dashed line represents chance performance (AUC = 0.5). AUC values with 95% CIs estimated using 1000 bootstrap resamples

Figure S2. Standardized Mean Differences (SMD) for baseline characteristics with notable imbalances between treatment groups.

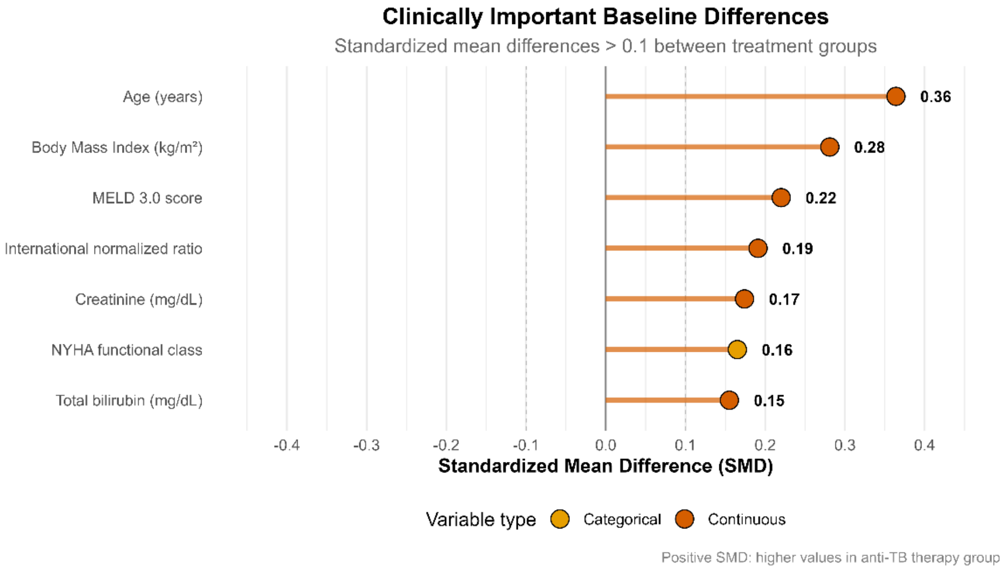

Standardized mean differences (SMD) comparing patients with (n = 139) and without (n = 51) preoperative anti-tuberculosis therapy. Positive values indicate higher means in the therapy group. Dashed lines mark the threshold for clinically meaningful imbalance (|SMD| > 0.1).

Figure S3. Proportional hazards assumption assessment for ICU discharge.

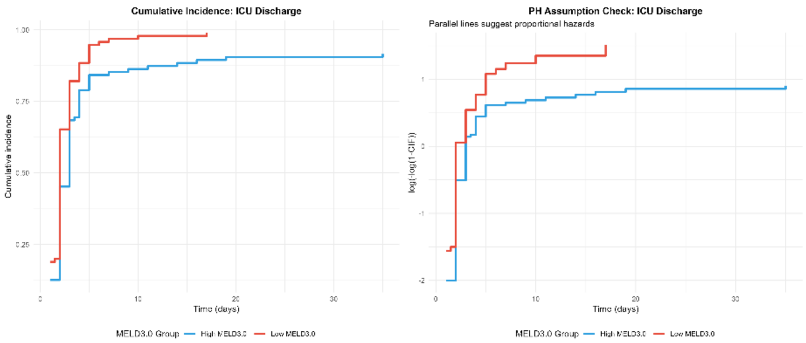

Log-log cumulative incidence plots stratified by MELD 3.0 group. Parallel curves suggest the proportional hazards assumption holds (Slope difference: 0.108).

Figure S4. Proportional hazards assumption assessment for postoperative discharge.

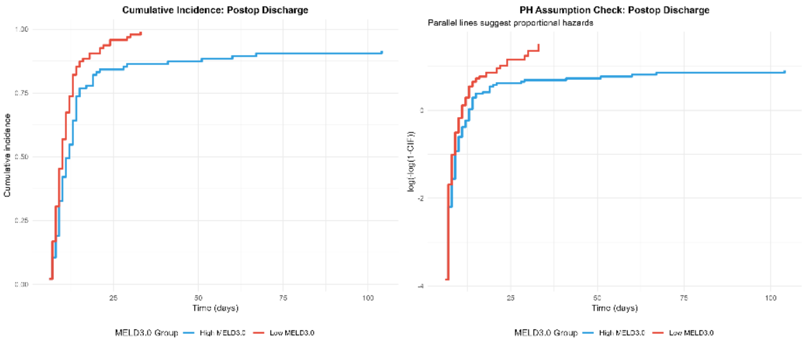

Log-log cumulative incidence plots illustrating good parallelism between MELD 3.0 groups (Slope difference: 0.094), supporting model validity.

Figure S5. Proportional hazards assumption assessment for total hospital discharge.

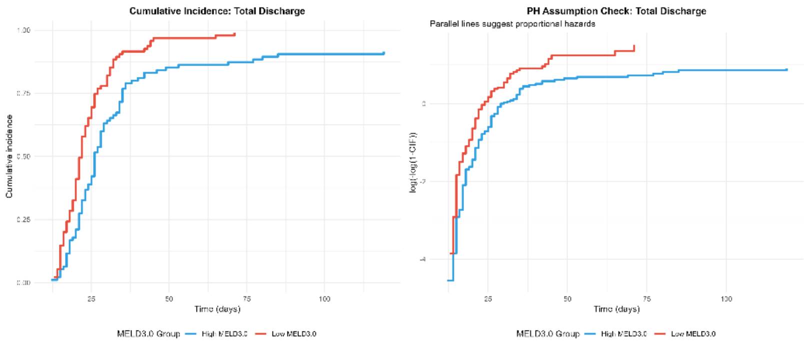

Log-log cumulative incidence plots showing excellent parallelism (Slope difference: 0.030), confirming the proportional hazards assumption for the primary endpoint.

Supplementary Table S1. Univariate associations between preoperative MELD 3.0 score and postoperative major adverse events

Outcome	Unadjusted OR (95% CI)	*p*-value
Operative mortality	1.21 (1.04–1.41)	0.013
Postoperative renal failure	1.18 (1.03–1.35)	0.015
Postoperative Low Cardiac Output Syndrome	1.03 (0.92–1.16)	0.559

Abbreviations: CI, confidence interval; OR, odds ratio.

Event rates: Operative mortality, 4.7% (9/190); Postoperative renal failure, 12.1% (23/190); Postoperative Low Cardiac Output Syndrome, 8.9% (17/190).

Supplementary Table S2. Multivariable Fine-Gray competing risks regression analysis with MELD 3.0 as a continuous variable

Outcome	Variable	Comparison	sHR	95% CI	*p*-value
ICU Discharge	MELD 3.0 score	Per 1-unit increase	0.905	0.865–0.947	<0.001
	Age	Per year increase	1.003	0.996–1.010	0.440
	Preoperative albumin	Per 1 g/dL increase	1.080	0.776–1.503	0.650
	Preoperative anti-TB therapy	Yes vs No	0.727	0.561–0.941	0.015
Postoperative Discharge	MELD 3.0 score	Per 1-unit increase	0.900	0.858–0.945	<0.001
	Age	Per year increase	0.995	0.987–1.003	0.200
	Preoperative albumin	Per 1 g/dL increase	1.527	1.039–2.244	0.031
	Preoperative anti-TB therapy	Yes vs No	0.675	0.514–0.886	0.005
Total Hospital Discharge	MELD 3.0 score	Per 1-unit increase	0.878	0.831–0.927	<0.001
	Age	Per year increase	0.993	0.984–1.002	0.130
	Preoperative albumin	Per 1 g/dL increase	1.820	1.237–2.678	0.002
	Preoperative anti-TB therapy	Yes vs No	0.724	0.531–0.987	0.041

All models were adjusted for the covariates listed. Analysis performed using Fine-Gray competing risks models with in-hospital death treated as a competing event. sHR = subdistribution hazard ratio; sHR < 1 indicates slower discharge for the comparison group. TB = tuberculosis.

Supplementary Table S3. Time-dependent AUC values at clinically relevant time points

Time (days)	Events (discharge)	Cumulative %	AUC	95% CI
15	19	10.0%	0.755	—
20	59	31.1%	0.715	—
21	73	38.4%	0.721	0.641–0.796
25	106	55.8%	0.704	—
30	139	73.2%	0.696	—
35	160	84.2%	0.682	—
45	171	90.0%	0.742	0.625–0.853

Time-dependent AUCs were calculated using the inverse probability of censoring weighting (IPCW) method implemented in the timeROC R package. The linear predictor from the multivariable Fine-Gray model was used as the marker. 95% confidence intervals were estimated using 1000 bootstrap resamples and are reported for the two representative time points (21 and 45 days). The 21-day time point represents the earliest point with sufficient events (n=73, 38.4%) for stable AUC estimation, while 45 days represents the optimal discriminative performance (n=171, 90.0%). Dashes indicate time points for which 95% CIs were not calculated.

Supplementary Table S4. Subgroup analysis: Multivariable competing risks model for time to total hospital discharge in patients without preoperative anti-tuberculosis therapy (n = 51).

Variable	Comparison	sHR	95% CI	*p*-value
MELD 3.0 score	High vs Low	0.809	0.464–1.409	0.453
Age	Per year increase	0.979	0.964–0.994	0.006
Preoperative albumin	Per 1 g/dL increase	4.039	2.040–7.997	<0.001

Model adjusted for age and preoperative albumin. Shr, subdistribution hazard ratio.

Supplementary Table S5. Interaction analysis: Association between MELD 3.0 score and total discharge time including interaction term

Variable	Comparison	sHR	95% CI	*p*-value
MELD 3.0 score	High vs Low	0.726	0.417–1.263	0.257
Anti-tuberculosis therapy	Yes vs No	0.844	0.512–1.391	0.506
MELD 3.0 × Anti-TB therapy	Interaction term	0.775	0.402–1.495	0.447
Age	Per year increase	0.992	0.984–1.001	0.077
Preoperative albumin	Per 1 g/dL increase	2.090	1.378–3.170	<0.001

sHR, subdistribution hazard ratio.

Supplementary Table S6. Bonferroni correction for multiple comparisons across three time-to-discharge outcomes

Outcome	Original p -value*	Bonferroni-adjusted p -value	Significant (α=0.017)
ICU discharge	0.0006	0.0018	Yes
Postoperative discharge	0.0034	0.0102	Yes
Total discharge	0.0007	0.0021	Yes

p -values are from the multivariable models (Table 2). The adjusted significance threshold after Bonferroni correction (k = 3) is α = 0.017.

Supplementary Table S7. Assessment of Proportional Hazards Assumption for Fine-Gray Competing Risks Models

Outcome	Slope Difference*	Parallelism Assessment	Cumulative Incidence Curves	PH Assumption Support
ICU Discharge	0.108	Slight deviation (near threshold 0.1)	Consistent separation without crossing	Reasonably supported
Postoperative Discharge	0.094	Good parallelism (below 0.1 threshold)	Consistent separation without crossing	Well supported
Total Hospital Discharge	0.030	Excellent parallelism (well below 0.1 threshold)	Consistent separation without crossing	Strongly supported

A slope difference of < 0.1 in log(-log) plots suggests no major violation of the proportionality assumption. PH, proportional hazards.
